# Linking cardiometabolic multimorbidity to depressive symptoms in the oldest-old: results from a cross-sectional study in Germany

**DOI:** 10.1186/s12889-025-22964-1

**Published:** 2025-05-09

**Authors:** Verena Maschke, Valerie Lohner, Ute Mons

**Affiliations:** Department of Cardiology, Faculty of Medicine and University Hospital Cologne, Cardiovascular Epidemiology of Aging, University of Cologne, Kerpener Straße 62, Cologne, 50937 Germany

**Keywords:** Depression, Cardiometabolic multimorbidity, Psychocardiology, Psychogeriatrics

## Abstract

**Background:**

Depression often accompanies cardiometabolic multimorbidity (CMM), but it remains unclear whether this association persists in very old people. Hence, we examined the link between CMM and depressive symptoms in an oldest-old population.

**Methods:**

Using cross-sectional data from a representative sample of individuals aged 80 years and older in North Rhine-Westphalia, Germany (*N* = 1,863), we constructed an additive disease index covering seven cardiometabolic diseases (CMDs): myocardial infarction, heart failure, hypertension, stroke, diabetes mellitus, kidney disease, and obesity. Depressive symptoms were assessed using the short form of the Depression in Old Age Scale (0 to 4 points). We employed multivariable linear regression models to study associations of CMD index (0, 1, 2, ≥ 3 CMDs) and CMD count (0 to 7 diseases) with depressive symptoms, adjusting for age, sex, socio-economic index, respiratory and pulmonary disease, cancer, and liver disease.

**Results:**

Participants had a mean depressive symptom score of 0.94, and 44% reported two or more CMDs. Heart failure, hypertension, stroke, and obesity were each individually associated with more depressive symptoms. Participants with two (β = 0.30; 95%-CI: 0.12–0.48), and three or more CMDs (β = 0.40; 95%-CI: 0.18–0.62) showed higher depressive symptoms compared to those with no CMD, i.e., each additional CMD was associated with a 0.30-unit or 0.40-unit increase in depressive symptoms, respectively. We observed an additive dose–response association between CMD count and depressive symptoms (β = 0.16; 95%-CI: 0.09–0.23), slightly more pronounced for women (β = 0.19; 95%-CI: 0.10–0.29) than for men (β = 0.10, 95%-CI: 0.02–0.19).

**Conclusions:**

Individuals with CMM showed increased depressive symptomatology, indicating the need to address both physical and mental health in oldest-old individuals with high CMD burden. However, the cross-sectional study design prevents conclusions about causality and warrants further longitudinal studies.

**Supplementary Information:**

The online version contains supplementary material available at 10.1186/s12889-025-22964-1.

## Introduction

Depression among the oldest-old population, here defined as 80 years or above, poses a substantial public health challenge. Prevalence in this population is substantial; for example, a recent study suggested that about 40% of the oldest-old in Germany had probable depression [1]. Furthermore, depression in the oldest-old is characterized by atypical symptom presentation and unique risk factors when compared to younger age groups [2]. This population often has a higher burden of chronic health conditions, functional limitations, and social isolation, all of which contribute to the complexity of depression management [2]. In particular, older people with depression commonly have a high prevalence of cardiovascular and metabolic diseases, potentially leading to cardiometabolic multimorbidity (CMM) [3, 4].

CMM, defined as the coexistence of two or more cardiometabolic diseases (CMDs) such as heart failure, stroke, and type 2 diabetes, is one of the most common multimorbidity profiles and a growing public health concern [5, 6]. While individual CMDs are well-established comorbidities of depression, current research increasingly examined whether the cumulative burden of multiple CMDs is associated with depression. Recent studies found that individuals with existing CMM are at an increased risk of experiencing subsequent depression [7–10], and suggested an additive dose–response relationship between the number of CMDs and the risk of depression [7, 8]. Notably, emerging research suggests a bidirectional relationship between CMM and depression, where CMM not only increases the risk of depression, but depression itself appears to contribute to the development of CMM [11–15]. For example, Qiao et al. observed that people with depression were more likely to develop CMM over time than people without depression, with the effect being strongest when stroke was part of the cardiometabolic disease cluster [11]. In addition, Wang et al. found a dose–response relationship between the severity of depressive symptoms and incident CMM, indicating that an increase in depressive symptoms lead to a higher likelihood of developing CMM [12]. The bidirectional approach was adopted by Zhou et al., who observed that depression and CMM independently act as risk factors for one another, with lifestyle behaviors such as physical inactivity and poor diet potentially mediating this effect [16].

Despite these advancements in understanding the association between CMM and depression, there remains a notable gap in research concerning the oldest-old population. Most studies have focused on populations of middle or older age, leaving uncertainty regarding whether similar patterns hold true for those over 80 years of age. Thus, although oldest-old people are particularly susceptible to both CMDs and depression, this fast-growing age group remains largely underrepresented in present research on CMM, and particularly in studies investigating the association of CMM with depression. Our objective was to bridge this gap by examining the relationship between the count of CMDs and severity of depressive symptoms, specifically focusing on oldest-old people participating in a population-based cross-sectional study, and thus, expanding existing knowledge to this particularly vulnerable age-group. This research might help inform targeted screening strategies and integrated care approaches that address both cardiometabolic and mental health in the oldest-old.

## Methods

### Study population

This study utilized existing, publicly available cross-sectional data from the "Survey on Quality of Life and Subjective Well-being of the Very Old in North Rhine-Westphalia (NRW80 +)" [17], provided by GESIS – Leibniz Institute for the Social Sciences. This survey was conducted between August 2017 and February 2018 in North Rhine-Westphalia, the most populous federal state in Germany. People who had reached the age of 80 years before July 31, 2017 and were registered as having their main residence in North Rhine-Westphalia were eligible for this survey. From this population, a random sample was drawn from registration offices, with oversampling of men and individuals aged 85 years and older. Self-reported data were collected through face-to-face interviews with the study participants or a proxy, administered by trained interviewers. The final sample consisted of 1,863 individuals, with 176 (9.4%) interviews conducted via proxies. The response rate was 26.5%. Factors such as sex, age group and living arrangement were not significantly related to the likelihood of nonresponse. Further details regarding the methodology can be found elsewhere [18].

The NRW80 + survey has been approved by the ethics committee of the Medical Faculty of the University of Cologne, Germany (No. 17–169). Informed consent had been obtained from all participants or their legal representatives.

### Depressive symptoms

Depressive symptoms were assessed with the short form of the Depression in Old Age Scale (DIA-S4), a validated instrument for measuring depressive symptoms in older people [19]. The scale contains the following four items with binary response options (yes vs. no), referring to the last 14 days: (1) Do you have feelings of oppression?, (2) Do you struggle with motivation?, (3) Can you enjoy your life, even if you find some things more difficult?, and (4) Do you have to ponder a lot?. The sum score was calculated by adding the confirmed items, resulting in a sum score ranging from 0 to 4, with a higher score indicating more depressive symptoms. A cut-off of 1.5 points was used for sub-diagnostic but clinically relevant depressive mood [19].

### Cardiometabolic disease index

The NRW80 + survey assessed multimorbidity with an index covering 19 chronic diseases with binary response options, with interviewers asking for each of these diseases whether the participant currently receives treatment for it [20]. We selected all cardiovascular and metabolic diseases for construction of an additive cardiometabolic disease index: myocardial infarction, heart failure, hypertension, stroke, diabetes, and kidney disease. Additionally, we included obesity in the CMD index, defined as a Body-Mass-Index (BMI) ≥ 30. Obesity was considered as a metabolic disease as it is associated with metabolic dysregulation and systemic inflammation, and an increased risk for other CMDs [21]. Consequently, the disease index comprised seven CMDs. In addition to the continuous CMD index, we categorized the disease index into four groups: 0, 1, 2, and ≥ 3 CMDs, given the small sample size of people with four or more concurrent CMDs (*N* = 85).

### Covariates

Covariates included sex (woman or man), age group (80–84, 85–89, 90 years and older), the continuous socio-economic index by Ganzeboom et al., [22] and chronic respiratory and pulmonary disease, cancer, and liver disease, assessed with the multimorbidity index.

### Statistical analysis

We reported descriptive results with mean and standard deviation (SD), and analysed mean differences with two-tailed t-tests. For our main analyses, we first examined the association between each individual CMD and depressive symptoms using multivariable linear regression models. Secondly, we studied associations between the categorical CMD index (0, 1, 2, ≥ 3 CMDs) and depressive symptoms using a multivariable linear regression model. Thirdly, we assessed the potential additive dose–response relationship between continuous CMD index (0 to 7 CMDs) and depressive symptoms. Additionally, we explored whether sex moderated the associations of CMD index and depression (1) by incorporating interaction terms of sex with both the categorical and continuous CMD index, and (2) by stratifying the analyses by sex. To assess the robustness of our results, we conducted sensitivity analyses, which involved: (1) excluding each CMD individually from the CMD index, (2) excluding participants whose data were collected via proxies, and (3) using depressive mood as binary outcome in logistic regression models. All models were adjusted for age, sex (except for the sex-stratified analyses), socio-economic index, respiratory and pulmonary disease, cancer, and liver disease.

We applied pre-assigned weights [23] in all regression analyses, derived by design and adjustment weighting to account for the sampling design and non-respondents, thus ensuring the representativeness of our results. Statistical significance was defined as *p*< 0.05, with heteroscedasticity robust standard errors. All analyses were performed in R version 4.2.1 [24].

## Results

### Characteristics of the study population

**Fig. 1:**
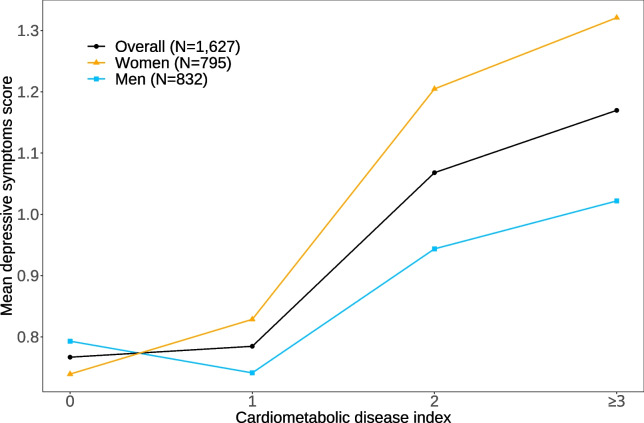
Mean depressive symptoms score according to the categorical cardiometabolic disease index

Detailed characteristics of the study population are provided in Table 1. Due to the sampling design, both sex and age groups were evenly distributed. Men had a higher mean socioeconomic index compared to women. The mean score of depressive symptoms was 1.01 for women and 0.87 for men, with a statistically significant difference between sexes (*p* = 0.008). About half of the study participants experienced at least one depressive symptom, with one in four men and one in three women meeting the criteria for depressive mood. The number of CMDs ranged from zero to a maximum of five concurrent CMDs. The mean number of CMDs was 1.46, and 44% of the population had CMM (i.e., two or more CMDs). As shown in Fig. 1, depressive symptoms increased with a higher CMD index, and we observed a steeper increase in women compared to men. The most prevalent CMD was hypertension and the most common CMD combination was hypertension with heart failure (See Supplementary Material A1, Additional File 1).

**Table 1:** Baseline characteristics of the study population

	**Overall (*N* = 1,863)**	**Men (*N* = 927)**	**Women (*N* = 936)**	**Missing values (n)**
**Age group, n (%)**				0
80–84 years	728 (39.1%)	384 (41.4%)	344 (36.8%)	
85–89 years	625 (33.5%)	299 (32.3%)	326 (34.8%)	
90 years and older	510 (27.4%)	244 (26.3%)	266 (28.4%)	
**Socio-economic index, mean (SD)**	42.66 (21.23)	47.73 (21.97)	37.50 (19.12)	60
**Multi-morbidity index, mean (SD)**	0.18 (0.12)	0.18 (0.12)	0.19 (0.13)	20
**Myocardial infarction, n (%)**	136 (7.4%)	99 (10.7%)	37 (4.0%)	20
**Heart failure, n (%)**	665 (36.1%)	319 (34.6%)	346 (37.5%)	20
**Hypertension, n (%)**	1,077 (58.4%)	527 (57.2%)	550 (59.7%)	20
**Stroke, n (%)**	143 (7.8%)	74 (8.0%)	69 (7.5%)	20
**Diabetes, n (%)**	293 (15.9%)	153 (16.6%)	140 (15.2%)	20
**Kidney disease, n (%)**	140 (7.6%)	77 (8.4%)	63 (6.8%)	20
**Obesity (BMI ≥ 30 kg/m**^**2**^**), n (%)**	223 (12.7%)	95 (10.6%)	128 (14.9%)	104
**Respiratory and pulmonary disease**	234 (12.7%)	119 (12.9%)	115 (12.5%)	20
**Cancer**	153 (8.3%)	101 (11.0%)	52 (5.6%)	20
**Liver disease**	24 (1.3%)	9 (1.0%)	15 (1.6%)	20
**Depressive symptoms, mean (SD)**	0.94 (1.13)	0.87 (1.06)	1.01 (1.19)	136
**Depressive symptoms, n (%)**				136
0	837 (48.5%)	426 (49.7%)	411 (47.3%)	
1	418 (24.2%)	221 (25.8%)	197 (22.7%)	
2	270 (15.6%)	133 (15.5%)	137 (15.8%)	
3	143 (8.3%)	55 (6.4%)	88 (10.1%)	
4	59 (3.4%)	23 (2.7%)	36 (4.1%)	
**Depressive mood (DIA-S ≥ 1.5), n (%)**	472 (27.3%)	211 (24.6%)	261 (30.0%)	136
**Continuous CMD index (0–7), mean (SD)**	1.46 (1.12)	1.47 (1.12)	1.45 (1.12)	114
**Categorized CMD index, n (%)**				114
0	362 (20.7%)	185 (20.7%)	177 (20.7%)	
1	618 (35.3%)	312 (34.9%)	306 (35.8%)	
2	475 (27.2%)	247 (27.6%)	228 (26.7%)	
≥ 3	294 (16.8%)	151 (16.9%)	143 (16.7%)	
**Interview with proxy, n (%)**	176 (9.4%)	65 (7.0%)	111 (11.9%)	0

*BMI* Body mass index, *CMD* Cardiometabolic disease, *DIA-S4* Depression in Old Age Scale, short form, *SD* Standard deviation

### Results from multivariate linear regression models

**Fig. 2:**
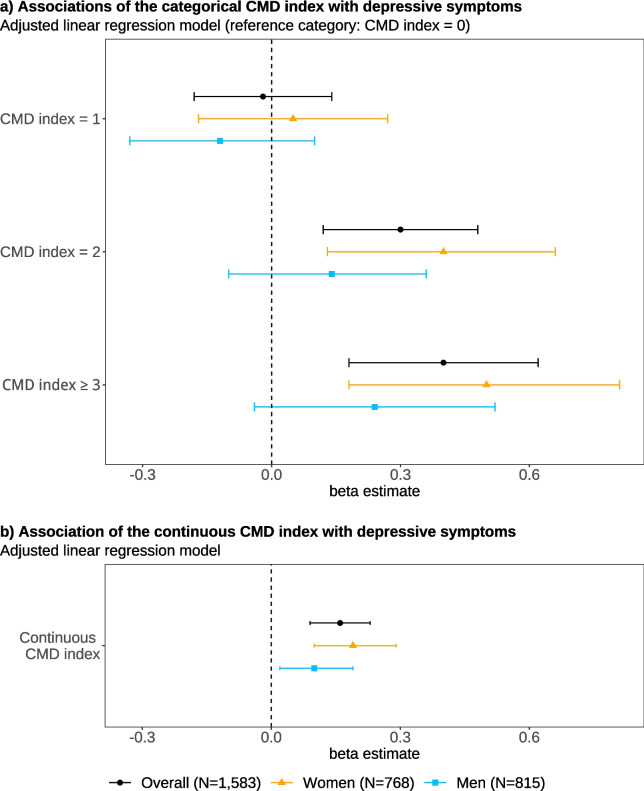
Associations of the CMD indices with depressive symptoms: results from linear regression (β with 95%-CI) Legend: Footnote: Standard errors are heteroscedasticity robust. Both models are adjusted for sex (except for the sex-stratified analyses), age, socio-economic status, respiratory and pulmonary disease, cancer, and liver disease. β—beta coefficient. CI—confidence interval. CMD—cardiometabolic disease. Ref.—reference category

The associations between individual CMDs and CMD indices with depressive symptoms are shown in Table 2. Among the included CMDs, heart failure, hypertension, stroke and obesity were individually associated with higher levels of depressive symptoms. When including the categorical CMD index, participants with two CMDs, and those with three or more CMDs showed an increase in depressive symptoms compared to those with no CMD. We observed a statistically significant additive dose–response relationship between the continuous CMD index and depressive symptoms, implying that a higher number of concurrent CMDs was linked to increased levels of depressive symptoms. Sex-stratified analyses revealed that the association between CMD index (both categorical and continuous) and depressive symptoms was more pronounced in women than in men. However, we found no statistically significant interactions by sex. The associations between CMD indices and depressive symptoms are visualized in Fig. 2.

**Table 2:** Associations of individual CMDs and CMD indices with depressive symptoms: results from linear regression models

*Individual CMDs*	Unadjusted estimates			Adjusted estimates^a^		
	β	95% CI	*p-value*	β	95% CI	*p-value*
Myocardial infarction	0.10	−0.14 – 0.33	0.428	0.12	−0.12 – 0.35	0.330
Heart failure	0.32	0.19–0.46	<.001	0.26	0.12–0.40	<.001
Hypertension	0.12	−0.01 – 0.25	0.059	0.13	0.01–0.25	0.042
Stroke	0.44	0.16–0.73	0.003	0.38	0.10–0.67	0.009
Diabetes	0.19	0.01–0.37	0.043	0.15	−0.04 – 0.33	0.114
Kidney disease	0.21	−0.02 – 0.45	0.077	0.13	−0.12 – 0.37	0.308
Obesity	0.24	0.04–0.43	0.016	0.20	0.01–0.40	0.036
*CMD indices*	Unadjusted estimates			Adjusted estimates^a^		
	β	95% CI	*p-value*	β	95% CI	*p-value*
**Overall**						
Categorical CMD index						
0 (Ref.)	0.00			0.00		
1	−0.00	−0.16 – 0.16	0.987	−0.02	−0.18 – 0.14	0.838
2	0.33	0.15–0.52	<.001	0.30	0.12–0.48	0.001
≥ 3	0.45	0.24–0.67	<.001	0.40	0.18–0.62	<.001
Continuous CMD index	0.18	0.11–0.24	<.001	0.16	0.09–0.23	<.001
N	1,627			1,583		
**Women**						
Categorical CMD index						
0 (Ref.)	0.00			0.00		
1	0.07	−0.14 – 0.29	0.507	0.05	−0.17 – 0.27	0.685
2	0.46	0.20–0.71	<.001	0.40	0.13–0.66	0.003
≥ 3	0.57	0.27–0.87	<.001	0.50	0.18–0.81	0.002
Continuous CMD index	0.22	0.13–0.31	<.001	0.19	0.10–0.29	<.001
N	795			768		
**Men**						
Categorical CMD index						
0 (Ref.)	0.00			0.00		
1	−0.13	−0.34 – 0.09	0.248	−0.12	−0.33 – 0.10	0.284
2	0.13	−0.09 – 0.36	0.246	0.14	−0.10 – 0.36	0.243
≥ 3	0.24	−0.05 – 0.53	0.101	0.24	−0.04 – 0.52	0.098
Continuous CMD index	0.11	0.02–0.19	0.016	0.10	0.02–0.19	0.016
N	832			815		

Standard errors are heteroscedasticity robust

*β* Beta coefficient, *CI* Confidence interval, *CMD* Cardiometabolic disease, *Ref.* Reference category

^a^Adjusted for sex (except for the sex-stratified analyses), age, socio-economic status, respiratory and pulmonary disease, cancer, and liver disease

### Non-responder analysis

We examined potential differences between study participant included in the final analysis (*N* = 1,583) and study participants excluded in final analysis due to missing data (*N* = 280). Across all missing data (*N* = 280), women in the age groups 85–89 years and 90 years and older had more missing values compared to other age groups (See Supplementary Table A2, Additional File 1). Study participants with a higher socioeconomic index were more likely to complete the DIA-S4. Apart from that, there was no difference between study participants with (*N* = 136) or without missing depressive symptom score (See Supplementary Table A3, Additional File 1). Study participants with missing data on the CMD index (*N* = 114) were more likely female, in the age groups 85–89 years and 90 years and older, had a higher depressive symptom score, and a lower socioeconomic status (See Supplementary Table A4, Additional File 1).

### Sensitivity analyses

Excluding each CMD individually from the CMD index, and excluding participants whose data were collected via proxies did not substantially alter the results (See Supplementary Figure A2 and Table A5, Additional File 1). Logistic regression models with depressive mood as the outcome yielded similar findings to the main analyses. The Odds Ratio (OR) was 1.43 (95% confidence interval (CI): 1.02–2.00) for two CMDs and 1.58 (95%-CI: 1.10–2.28) for three or more CMDs compared to no CMD. The OR for the continuous CMD index was 1.24 (95%-CI: 1.10–1.39). We also observed similar sex trends (See Supplementary Table A6, Additional File 1).

## Discussion

To the best of our knowledge, this is the first study examining the association between CMM and depressive symptoms in an oldest-old population. We found that compared to people free of CMDs, oldest-old people with two and three or more CMDs experienced more depressive symptoms and were at higher risk for depressive mood after adjusting for potential confounders. We observed a particularly sharp increase in depressive symptoms for people with two CMDs. Additionally, our analyses confirmed an additive dose–response relationship of the amount of concurrent CMDs with the magnitude of depressive symptoms. For women, these associations tended to be more pronounced, even though we found no statistically significant interaction effects.

Previous studies in middle-aged and older adults reported a significant increase in depression prevalence with each additional CMD, compared to no CMD, and a significant additive dose–response relationship between CMD count and depression prevalence [7–10, 16]. Our results are consistent with these findings and extend them to the oldest-old population. Interestingly, previous studies in younger populations indicated that depression is a risk factor for progression from a CMD-free status to CMM [11–16]. Overall, studies examining the association between CMM and depression used either a prospective [8, 10–12, 14–16], or a cross-sectional study design [7, 9, 13]. The mean number of included CMDs across studies was four and all studies defined CMM as two or more CMDs. Most studies assessed self-reported depression with a validated assessment instrument [8, 9, 11–16]. The assessment of CMDs varied across studies, including self-report [7–10, 12–16], clinical assessment [8, 12, 15] and ICD diagnoses [11, 14].

While the findings of these previous studies collectively imply a bidirectional relationship between depression and CMM, potential underlying mechanisms for this association are less understood. One explanation could be the heightened psychosocial distress experienced with CMM and related self-management activities, including polypharmacy and regular doctor visits, as well as exacerbated CMD symptoms and progression, including functional and neurocognitive impairments and adverse events [25]. Additionally, lifestyle factors such as sedentary behaviour and unhealthy diet are symptoms of depression as well as established risk factors for CMM [26, 27], and both depression and the onset and progression of CMDs are linked to pathophysiological factors such as inflammatory and hypothalamic–pituitary–adrenal axis dysregulation [28, 29]. Particularly in people experiencing an atypical, energy-related depressive symptom combination, such as increased appetite, hypersomnia, or loss of energy, recent research found elevated inflammatory marker levels, indicating shared immuno-metabolic pathways between depression and CMDs [30].

We found a higher prevalence of depressive mood in women with CMM, compared to men, aligning with other studies [11, 14, 15]. Three previous studies observed a significant interaction between sex and CMD index in older people, of which two identified stronger associations in women and one a stronger association in men [7, 12, 14]. Qiao et al. support our non-significant trend of a more pronounced association in women, while Qin et al. found a more pronounced association for men [11, 15]. Potential sex differences in the association between CMM and depression may be driven by several factors. Women’s stronger inflammatory activity may lead to a more gradual increase in depressive symptoms as CMDs accumulate [31]. On a psychosocial level, men tend towards more avoidance-based coping strategies, which may mask depressive symptoms until CMDs have accumulated to a critical level [32]. This could result in a more pronounced, steeper increase in depression for men when multiple CMDs are present. Additionally, women are more likely to seek healthcare and report symptoms, which may result in an earlier diagnosis and a more linear relationship between CMDs and depression. Thus, findings of a potentially amplifying sex-specific effect on the association between CMM and depression remain ambiguous and should be subject of further research.

Generally, our results on the association between CMM and depressive symptoms in oldest-old people emphasize the importance of integrated physical and mental health treatment among people with high CMD burden in this population. Effective implementation of integrated care could be guided by established models, such as collaborative care approaches, which emphasize multidisciplinary coordination between general practitioners, cardiologists, geriatricians, mental health specialists, and social care providers, and which have shown promising results in jointly managing CMDs and depression in older people [33–35]. A first step toward improved care might include the systematic implementation of routine screening for depressive symptoms in patients with CMM in primary and specialist care settings, ensuring early detection and intervention to prevent adverse effects on treatment adherence and prognosis. Similarly, to reduce morbidity and disability arising from cardiometabolic conditions, health professionals should proactively monitor CMDs in individuals with depression and adopt a comprehensive care strategy that jointly addresses shared risk factors, such as inflammation, physical inactivity, and social isolation. Future research should explore how integrated care models can be tailored to the unique needs of the oldest-old population to enhance feasibility and effectiveness.

Furthermore, preventive lifestyle factors may mitigate the link between CMM and depression. Especially physical activity and social support play key roles in older people, as both inactivity and loneliness independently increase the risk of developing CMM [36, 37] and depressive symptoms [38]. Additionally, Herbolsheimer et al. observed that old people who felt socially isolated performed less out-of-home physical activity, which in turn was associated with a greater risk for depression [39]. People with at least one CMD were half as likely to be hospitalised for a mental disorder when being physically active compared to those being inactive [40]. In people with both CMM and depression, being physically active was associated with 6.81 additional life years [41]. However, a study among Chilean adults found the strongest association with cardiometabolic risk factors among subjects that have depressive symptoms but are physically active [42]. Future high-quality research on physical activity and social support as potential protective factors of both CMM and depression is needed.

Several limitations of our study should be acknowledged. First, our results are based on cross-sectional data, which preclude us from drawing conclusions about causality of associations. Second, we rely on self-reported data, which introduces the potential for misclassification, e.g., due to recall or social desirability bias. Additionally, CMDs (except obesity) were defined as self-reported treatment status, which could potentially lead to an underestimation of disease prevalence. This limitation could result in a more conservative estimate that attenuates rather than strengthens the observed associations between CMM and depressive symptoms. In 9.4% of cases, proxies answered on behalf of participants, which helped include individuals who might otherwise be unable to participate, thereby improving the representativeness of this often underrepresented population. However, proxy reporting may introduce bias if variables are systematically misreported, though sensitivity analyses showed no difference in results with or without proxies. The overall response rate of 26.5% may introduce selection bias, as it can be assumed that people with high depressive symptoms or severe multimorbidity tended to be less likely to participate. However, sociodemographic factors were not significantly related to the likelihood of nonresponse [18]. Additionally, we applied adjustment weighting to account for non-respondents [23]. Item non-response (i.e., missing values) for the CMD-index could have also biased our estimates, as those with missing values were more likely female, older than 85 years, had a higher depressive symptom score, and a lower socioeconomic status. This might have led to an underestimation of the association between CMM and depressive symptoms. However, the fact that missing values were not missing at random ruled out multiple imputation of missing values.

On the other hand, the main strength of this study is the use of a comprehensive representative sample of oldest-old people, who were often underrepresented in previous research in this area. Additionally, depressive symptoms were assessed using a screening instrument specifically developed for people at old age, and we treated depressive symptoms as a continuous outcome to preserve maximum variability for analyses. Furthermore, sensitivity analyses corroborated the robustness of the main results.

## Conclusions

The main finding of this study is the extension of the positive association between CMM and depression to oldest-old people. Additionally, we found a significant additive dose-repose relationship, indicating increased depressive symptomatology in people with higher CMD burden. Given that nearly half of the study participants experienced CMM, with almost a third showing signs of a depressive mood, our findings highlight the profound public health challenge presented by the co-morbidity of CMM and depression in aging populations. A holistic approach to preventing and managing depressive symptoms in oldest-old people is essential, focusing on targeted preventive and therapeutic measures for CMM, rather than solely addressing individual CMDs. However, sustainable improvements in care will require not only individualized interventions but also structural adaptations, including policies that facilitate integrated, multidisciplinary health care.

Future prospective studies based on various data types, such as clinical or routine data, considering oldest-old populations are needed. Longitudinal cohort studies could help establish causal relationships, biomarker analyses may shed light on biological mechanisms, and intervention trials could assess the effectiveness of integrated treatment approaches. Additionally, efforts to disentangle underlying mechanisms are necessary, to eventually derive clinically relevant recommendations for the aligned treatment of depression and CMM.

## Supplementary Information

Supplementary Material 1.

## Data Availability

The data analysed during this study are openly available in GESIS, Cologne at 10.4232/1.13978, reference number ZA7558.

## Abbreviations

BMI: Body-Mass-Index
CI: Confidence interval
CMD: Cardiometabolic disease
CMM: Cardiometabolic multimorbidity
DIA-S4: Short form of the Depression in Old Age Scale
NRW80 +: Survey on Quality of Life and Subjective Well-being of the Very Old in North Rhine-Westphalia
OR: Odds ratio
Ref.: Reference category
SD: Standard deviation
β: Beta coefficient
