# Supplementary material for: Linking cardiometabolic multimorbidity to depressive symptoms in the oldest-old: results from a cross-sectional study in Germany
Source: BMC Public Health. 2025 May 9;25:1720. doi: 10.1186/s12889-025-22964-1 (PMC12063236; doi:10.1186/s12889-025-22964-1)
Supplement: Supplementary file 1 — Supplementary Material 1. [file 12889_2025_22964_MOESM1_ESM.docx]

**SUPPLEMENTAL ONLINE MATERIALS**

**Linking cardiometabolic multimorbidity to depressive symptoms in oldest-old people: results from a cross-sectional study in Germany**

Verena Maschke^1^, Valerie Lohner^1^, Ute Mons^1^

Affiliations:

^1^Cardiovascular Epidemiology of Aging, Department of Cardiology, Faculty of Medicine and University Hospital Cologne, University of Cologne, Germany.

**Table of contents**

[**A1. Descriptive analyses** 3](#_Toc172534219)

[**Table A1** The mean of depressive symptoms with standard deviation (SD) and the prevalence of depressive mood with 95% confidence interval (CI) according to individual cardiometabolic diseases (CMD) and categorical CMD index 3](#_Toc172534220)

[**Figure A1** The ten most common cardiometabolic disease (CMD) combinations. 4](#_Toc172534221)

[**A2. Sensitivity analyses** 5](#_Toc172534222)

[**Figure A2** Associations of cardiometabolic disease (CMD) indices excluding each CMD individually with depressive symptoms: results from linear regression models (beta estimates with 95% confidence intervals). 5](#_Toc172534223)

[**Table A2** Associations of individual cardiometabolic diseases (CMDs) and CMD indices with depressive symptoms, excluding proxy interviews: results from linear regression models. 6](#_Toc172534224)

[**Table A3** Associations of individual cardiometabolic diseases (CMDs) and CMD indices with depressive mood: results from logistic regression models. 7](#_Toc172534225)

# **A1. Descriptive analyses**

**Table A1** The mean of depressive symptoms with standard deviation (SD) and the prevalence of depressive mood with 95% confidence interval (CI) according to individual cardiometabolic diseases (CMD) and categorical CMD index.

|  |  | Depressive symptoms^a^ | | Depressive mood^b^ | |
| --- | --- | --- | --- | --- | --- |
|  | N | Mean | SD | n (%) | 95% CI |
| Myocardial infarction |  |  |  |  |  |
| no | 1581 | 0.93 | 1.13 | 429 (27.1) | 25.0 – 29.4 |
| yes | 128 | 1.05 | 1.09 | 36 (28.1) | 21.1 – 36.5 |
| Heart failure |  |  |  |  |  |
| no | 1102 | 0.85 | 1.09 | 276 (25.0) | 22.6 – 27.7 |
| yes | 607 | 1.10 | 1.18 | 189 (31.1) | 27.6 – 35.0 |
| Hypertension |  |  |  |  |  |
| no | 710 | 0.89 | 1.14 | 184 (26.0) | 22.8 – 29.3 |
| yes | 999 | 0.97 | 1.11 | 281 (28.1) | 25.4 – 31.0 |
| Stroke |  |  |  |  |  |
| no | 1576 | 0.90 | 1.10 | 409 (25.6) | 23.8 – 28.2 |
| yes | 133 | 1.35 | 1.36 | 56 (42.1) | 34.1 – 50.6 |
| Diabetes |  |  |  |  |  |
| no | 1438 | 0.90 | 1.11 | 376 (26.1) | 23.9 – 28.5 |
| yes | 271 | 1.11 | 1.24 | 89 (32.8) | 27.5 – 38.6 |
| Kidney disease |  |  |  |  |  |
| no | 1582 | 0.92 | 1.13 | 426 (27.0) | 24.8 – 29.2 |
| yes | 127 | 1.12 | 1.14 | 39 (30.7) | 23.4 – 39.2 |
| Obesity |  |  |  |  |  |
| no | 1426 | 0.90 | 1.11 | 371 (26.0) | 23.8 – 28.4 |
| yes | 210 | 1.09 | 1.14 | 66 (31.4) | 25.5 – 38.0 |
| Categorical CMD index |  |  |  |  |  |
| 0 | 339 | 0.77 | 1.06 | 80 (23.6) | 19.4 – 28.4 |
| 1 | 576 | 0.78 | 1.04 | 124 (21.5) | 18.4 – 25.1 |
| 2 | 441 | 1.07 | 1.16 | 139 (31.5) | 27.4 – 36.0 |
| ≥ 3 | 271 | 1.17 | 1.21 | 90 (33.2) | 27.9 – 39.0 |

^a^4-item short form of depression in old age (DIA-S4, range: 0-4 points). ^b^DIA-S4 ≥ 1.5.

## **Figure A1** The ten most common cardiometabolic disease (CMD) combinations.


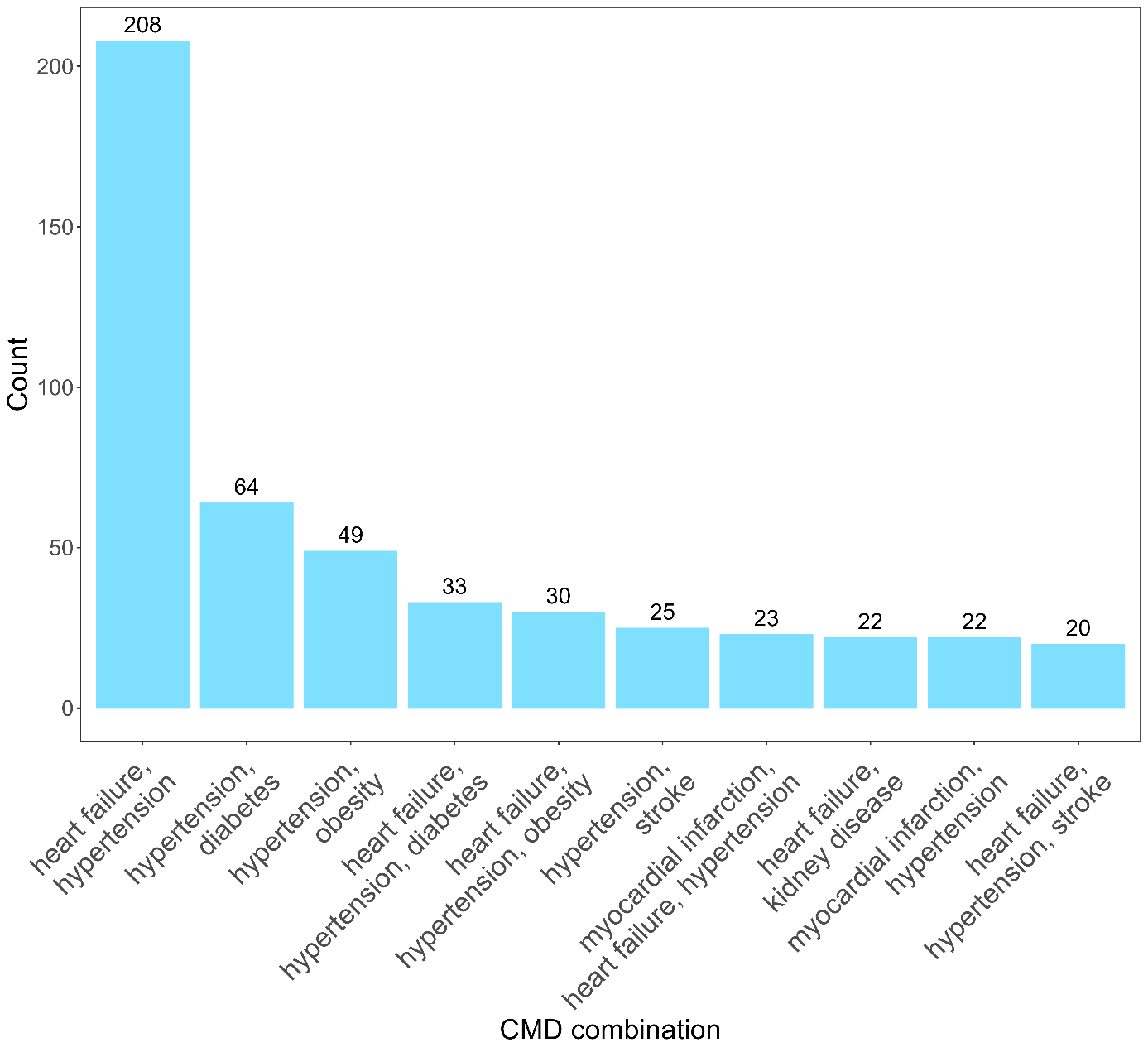


# **A2. Analysis of missing data**

## **Table A2** Comparison of study participants with missing data for the final analyses (All missing values).

|  | **Missing values**  **(N = 280)** | **Included**  **(N = 1,583)** | **p value** | **Statistical test** |
| --- | --- | --- | --- | --- |
| **Sex, n (%)** |  |  | 0.001 | Chi-square test |
| Men | 112 (40.0) | 815 (51.5) |  |  |
| Women | 168 (60.0) | 768 (48.5) |  |  |
| **Age group, n (%)** |  |  | <0.001 | Chi-square test |
| 80 – 84 years | 78 (27.9) | 650 (41.1) |  |  |
| 85 – 89 years | 103 (36.8) | 522 (33.0 |  |  |
| 90 years and older | 99 (35.4) | 411 (26.0) |  |  |

## **Table A3** Comparison of study participants with missing data for the final analyses (Missing values of DIA-S4).

|  | **Missing values**  **(N = 136)** | **Included**  **(N = 1,727)** | **p value** | **Statistical test** |
| --- | --- | --- | --- | --- |
|  | 136 | 1,727 |  |  |
| **Sex, n (%)** |  |  | 0.883 | Chi-square test |
| Men | 69 (50.7) | 858 (49.7) |  |  |
| Women | 67 (49.3) | 869 (50.3) |  |  |
| **Age group, n (%)** |  |  | 0.360 | Chi-square test |
| 80 – 84 years | 46 (33.8) | 682 (39.5) |  |  |
| 85 – 89 years | 47 (34.6) | 578 (33.5) |  |  |
| 90 years and older | 43 (31.6) | 467 (27.0) |  |  |
| **Categorized CMD index, n (%)** |  |  | 0.897 | Chi-square test |
| 0 | 23 (18.9) | 339 (20.8) |  |  |
| 1 | 42 (34.4) | 576 (35.4) |  |  |
| 2 | 34 (27.9) | 441 (27.1) |  |  |
| ≥ 3 | 23 (18.9) | 271 (16.7) |  |  |
| **Respiratory and pulmonary disease, n (%)** |  |  | 0.896 | Chi-square test |
| No | 116 (86.6) | 1,493 (87.4) |  |  |
| Yes | 18 (13.4) | 216 (12.6) |  |  |
| **Cancer, n (%)** |  |  | 0.440 | Chi-square test |
| No | 120 (89.6) | 1,570 (91.9) |  |  |
| Yes | 14 (10.4) | 139 (8.1) |  |  |
| **Liver disease, n (%)** |  |  | 0.325 | Chi-square test |
| No | 134 (100.0) | 1,685 (98.6) |  |  |
| yes | 0 (0.0) | 24 (1.4) |  |  |
| **Socio-economic index, mean (SD)** | 39.13 (19.24) | 42.93 (21.36) | 0.048 | t-test |

## **Table A4** Comparison of study participants with missing data for the final analyses (Missing values of the CMD index).

|  | **Missing values**  **(N = 114)** | **Included**  **(N = 1,749)** | **p value** | **Statistical test** |
| --- | --- | --- | --- | --- |
| **Sex, n (%)** |  |  | <0.001 | Chi-square test |
| Men | 32 (28.1) | 895 (51.2) |  |  |
| Women | 82 (71.9) | 854 (48.8) |  |  |
| **Age group, n (%)** |  |  | <0.001 | Chi-square test |
| 80 – 84 years | 26 (22.8) | 702 (40.1) |  |  |
| 85 – 89 years | 43 (37.7) | 582 (33.3) |  |  |
| 90 years and older | 45 (39.5) | 465 (26.6) |  |  |
| **Depressive symptoms, mean (SD)** | 1.23 (1.32) | 0.92 (1.12) | 0.008 | t-test |
| **Respiratory and pulmonary disease, n (%)** |  |  | 0.648 | Chi-square test |
| No | 84 (89.4) | 1,525 (87.2) |  |  |
| Yes | 10 (10.6) | 224 (12.8) |  |  |
| **Cancer, n (%)** |  |  | 0.907 | Chi-square test |
| No | 87 (92.6) | 1,603 (91.7) |  |  |
| Yes | 7 (7.4) | 146 (8.3) |  |  |
| **Liver disease, n (%)** |  |  | 1.000 | Chi-square test |
| No | 93 (98.9) | 1,726 (98.7) |  |  |
| yes | 1 (1.1) | 23 (1.3) |  |  |
| **Socio-economic index, mean (SD)** | 35.40 (19.20) | 43.09 (21.27) | <0.001 | t-test |

# **A3. Sensitivity analyses**

## **Figure A2** Associations of cardiometabolic disease (CMD) indices excluding each CMD individually with depressive symptoms: results from linear regression models (beta estimates with 95% confidence intervals).


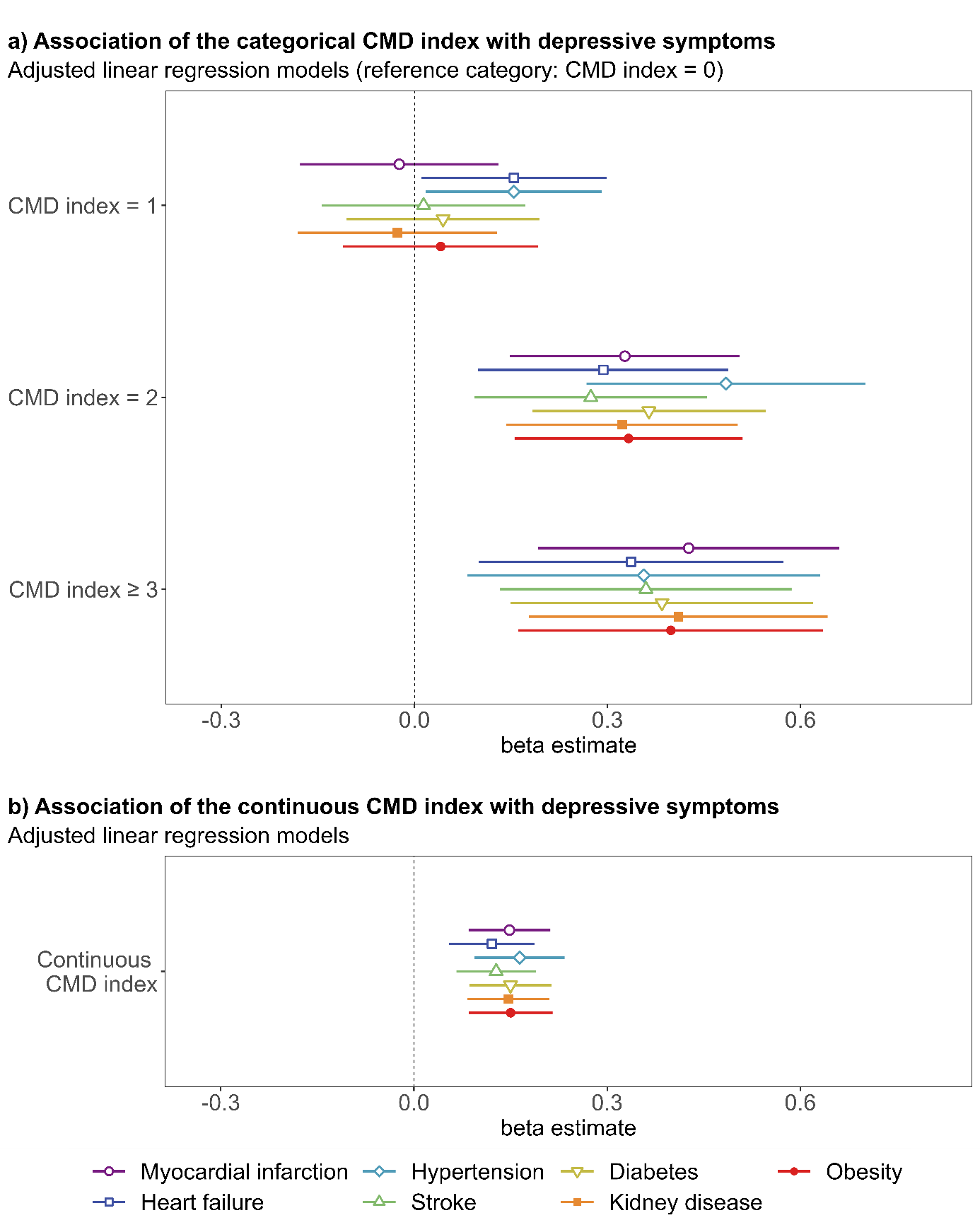


## **Table A5** Associations of individual cardiometabolic diseases (CMDs) and CMD indices with depressive symptoms, excluding proxy interviews: results from linear regression models.

| *Individual CMDs* | Unadjusted estimates | | |  | Adjusted estimates^a^ | | |
| --- | --- | --- | --- | --- | --- | --- | --- |
|  | β | 95% CI | p-value |  | β | 95% CI | p-value |
| Myocardial infarction | 0.08 | -0.15 – 0.31 | 0.500 |  | 0.08 | -0.15 – 0.32 | 0.483 |
| Heart failure | 0.32 | 0.18 – 0.46 | <.001 |  | 0.28 | 0.14 – 0.42 | <.001 |
| Hypertension | 0.11 | -0.02 – 0.24 | 0.098 |  | 0.11 | -0.01 – 0.24 | 0.078 |
| Stroke | 0.42 | 0.11 – 0.73 | 0.009 |  | 0.35 | 0.03 – 0.66 | 0.032 |
| Diabetes | 0.21 | 0.03 – 0.40 | 0.026 |  | 0.17 | -0.01 – 0.36 | 0.067 |
| Kidney disease | 0.25 | -0.00 – 0.49 | 0.053 |  | 0.18 | -0.08 – 0.43 | 0.174 |
| Obesity | 0.26 | 0.07 – 0.45 | 0.009 |  | 0.22 | 0.03 – 0.41 | 0.025 |
| *CMD indices* | Unadjusted estimates | | |  | Adjusted estimates^a^ | | |
|  | β | 95% CI | p-value |  | β | 95% CI | p-value |
| **Overall** |  |  |  |  |  |  |  |
| Categorical CMD index |  |  |  |  |  |  |  |
| 0 (Ref.) |  |  |  |  |  |  |  |
| 1 | 0.04 | -0.12 – 0.20 | 0.636 |  | 0.02 | -0.14 – 0.18 | 0.788 |
| 2 | 0.36 | 0.18 – 0.54 | <.001 |  | 0.32 | 0.14 – 0.50 | 0.001 |
| ≥ 3 | 0.48 | 0.26 – 0.70 | <.001 |  | 0.42 | 0.20 – 0.65 | <.001 |
| Continuous CMD index | 0.18 | 0.12 – 0.25 | <.001 |  | 0.16 | 0.10 – 0.23 | <.001 |
| N | 1,496 | | |  | 1,456 | | |
| **Women** |  |  |  |  |  |  |  |
| Categorical CMD index |  |  |  |  |  |  |  |
| 0 (Ref.) |  |  |  |  |  |  |  |
| 1 | 0.12 | -0.10 – 0.35 | 0.271 |  | 0.09 | -0.13 – 0.32 | 0.416 |
| 2 | 0.48 | 0.22 – 0.74 | <.001 |  | 0.41 | 0.14 – 0.67 | 0.003 |
| ≥ 3 | 0.61 | 0.30 – 0.92 | <.001 |  | 0.52 | 0.19 – 0.84 | 0.002 |
| Continuous CMD index | 0.23 | 0.13 – 0.32 | <.001 |  | 0.19 | 0.09 – 0.29 | <.001 |
| N | 709 | | |  | 687 | | |
| **Men** |  |  |  |  |  |  |  |
| Categorical CMD index |  |  |  |  |  |  |  |
| 0 (Ref.) |  |  |  |  |  |  |  |
| 1 | -0.10 | -0.31 – 0.11 | 0.356 |  | -0.09 | -0.31 – 0.12 | 0.398 |
| 2 | 0.17 | -0.05 – 0.40 | 0.133 |  | 0.17 | -0.06 – 0.40 | 0.149 |
| ≥ 3 | 0.25 | -0.03 – 0.54 | 0.078 |  | 0.26 | -0.03 – 0.54 | 0.077 |
| Continuous CMD index | 0.11 | 0.03 – 0.20 | 0.010 |  | 0.11 | 0.03 – 0.19 | 0.011 |
| N | 787 | | |  | 770 | | |

Footnote: Standard errors are heteroscedasticity robust. β - beta coefficient. CI - confidence interval. Ref. - reference category.

^a^Adjusted for sex (except for the sex-stratified analyses), age, socio-economic status, respiratory and pulmonary disease, cancer, and liver disease.

## **Table A6** Associations of individual cardiometabolic diseases (CMDs) and CMD indices with depressive mood: results from logistic regression models.

| *Individual CMDs* | Unadjusted estimates | | |  | Adjusted estimates^a^ | | |
| --- | --- | --- | --- | --- | --- | --- | --- |
|  | OR | 95% CI | p-value |  | OR | 95% CI | p-value |
| Myocardial infarction | 1.08 | 0.73 – 1.61 | 0.693 |  | 1.14 | 0.75– 1.73 | 0.537 |
| Heart failure | 1.57 | 1.26 – 1.96 | <.001 |  | 1.46 | 1.16 – 1.84 | 0.001 |
| Hypertension | 1.18 | 0.95 – 1.47 | 0.144 |  | 1.22 | 0.97 – 1.54 | 0.083 |
| Stroke | 2.03 | 1.41 – 2.90 | <.001 |  | 1.85 | 1.27 – 2.70 | 0.001 |
| Diabetes | 1.30 | 0.98 – 1.72 | 0.067 |  | 1.20 | 0.89 – 1.61 | 0.230 |
| Kidney disease | 1.23 | 0.83 – 1.83 | 0.296 |  | 1.07 | 0.70 – 1.63 | 0.749 |
| Obesity | 1.31 | 0.98 – 1.75 | 0.065 |  | 1.22 | 0.90 – 1.66 | 0.200 |
| *CMD indices* | Unadjusted estimates | | |  | Adjusted estimates^a^ | | |
|  | OR | 95% CI | p-value |  | OR | 95% CI | p-value |
| **Overall** |  |  |  |  |  |  |  |
| Categorical CMD index |  |  |  |  |  |  |  |
| 0 (Ref.) | 0.00 |  |  |  | 0.00 |  |  |
| 1 | 0.85 | 0.61 - 1.17 | 0.310 |  | 0.84 | 0.60 - 1.17 | 0.308 |
| 2 | 1.49 | 1.07 - 2.06 | 0.017 |  | 1.42 | 1.02 - 2.05 | 0.040 |
| ≥ 3 | 1.72 | 1.22 - 2.44 | 0.002 |  | 1.58 | 1.10 - 2.28 | 0.014 |
| Continuous CMD index | 1.27 | 1.14 – 1.42 | <.001 |  | 1.23 | 1.10 – 1.39 | <.001 |
| N | 1,627 | | |  | 1,583 | | |
| **Women** |  |  |  |  |  |  |  |
| Categorical CMD index |  |  |  |  |  |  |  |
| 0 (Ref.) | 0.00 |  |  |  | 0.00 |  |  |
| 1 | 1.01 | 0.64 – 1.59 | 0.973 |  | 0.99 | 0.65 – 1.50 | 0.960 |
| 2 | 1.68 | 1.06 – 2.67 | 0.028 |  | 1.55 | 1.01 – 2.37 | 0.043 |
| ≥ 3 | 2.11 | 1.29 – 3.45 | 0.003 |  | 1.84 | 1.16 – 2.91 | 0.009 |
| Continuous CMD index | 1.34 | 1.15 – 1.56 | <.001 |  | 1.27 | 1.10 – 1.47 | 0.001 |
| N | 795 | | |  | 768 | | |
| **Men** |  |  |  |  |  |  |  |
| Categorical CMD index |  |  |  |  |  |  |  |
| 0 (Ref.) | 0.00 |  |  |  | 0.00 |  |  |
| 1 | 0.60 | 0.38 – 0.97 | 0.036 |  | 0.62 | 0.35 – 1.10 | 0.101 |
| 2 | 1.20 | 0.76 – 1.90 | 0.432 |  | 1.22 | 0.70 – 2.13 | 0.477 |
| ≥ 3 | 1.15 | 0.68 – 1.91 | 0.607 |  | 1.18 | 0.63 – 2.20 | 0.606 |
| Continuous CMD index | 1.15 | 0.97 – 1.35 | 0.102 |  | 1.16 | 0.95 – 1.41 | 0.154 |
| N | 832 | | |  | 815 | | |

Footnote: CI - confidence interval. OR - Odds Ratio. Ref. - reference category.

^a^Adjusted for sex (except for the sex-stratified analyses), age, socio-economic status, respiratory and pulmonary disease, cancer, liver disease.
